# Changes in Weight, Waist Circumference and Compensatory Responses with Different Doses of Exercise among Sedentary, Overweight Postmenopausal Women

**DOI:** 10.1371/journal.pone.0004515

**Published:** 2009-02-18

**Authors:** Timothy S. Church, Corby K. Martin, Angela M. Thompson, Conrad P. Earnest, Catherine R. Mikus, Steven N. Blair

**Affiliations:** 1 Pennington Biomedical Research Center, Louisiana State University System, Baton Rouge, Louisiana, United States of America; 2 Department of Nutrition and Exercise Physiology, University of Missouri, Columbia, Missouri, United States of America; 3 Arnold School of Public Health, University of South Carolina, Columbia, South Carolina, United States of America; Institute of Preventive Medicine, Denmark

## Abstract

**Background:**

It has been suggested that exercise training results in compensatory mechanisms that attenuate weight loss. However, this has only been examined with large doses of exercise. The goal of this analysis was to examine actual weight loss compared to predicted weight loss (compensation) across different doses of exercise in a controlled trial of sedentary, overweight or obese postmenopausal women (n = 411).

**Methodology/Principal Findings:**

Participants were randomized to a non-exercise control (n = 94) or 1 of 3 exercise groups; exercise energy expenditure of 4 (n = 139), 8 (n = 85), or 12 (n = 93) kcal/kg/week (KKW). Training intensity was set at the heart rate associated with 50% of each woman's peak VO_2_ and the intervention period was 6 months. All exercise was supervised. The main outcomes were actual weight loss, predicted weight loss (exercise energy expenditure/ 7700 kcal per kg), compensation (actual minus predicted weight loss) and waist circumference. The study sample had a mean (SD) age 57.2 (6.3) years, BMI of 31.7 (3.8) kg/m^2^, and was 63.5% Caucasian. The adherence to the intervention was >99% in all exercise groups. The mean (95% CI) weight loss in the 4, 8 and 12 KKW groups was −1.4 (−2.0, −0.8), −2.1 (−2.9, −1.4) and −1.5 (−2.2, −0.8) kg, respectively. In the 4 and 8 KKW groups the actual weight loss closely matched the predicted weight loss of −1.0 and −2.0 kg, respectively, resulting in no significant compensation. In the 12 KKW group the actual weight loss was less than the predicted weight loss (−2.7 kg) resulting in 1.2 (0.5, 1.9) kg of compensation (P<0.05 compared to 4 and 8 KKW groups). All exercise groups had a significant reduction in waist circumference which was independent of changes in weight.

**Conclusion:**

In this study of previously sedentary, overweight or obese, postmenopausal women we observed no difference in the actual and predicted weight loss with 4 and 8 KKW of exercise (72 and 136 minutes respectively), while the 12 KKW (194 minutes) produced only about half of the predicted weight loss. However, all exercise groups had a significant reduction in waist circumference which was independent of changes in weight.

**Trial Registration:**

ClinicalTrials.gov NCT 00011193

## Introduction

The increasing prevalence of overweight, obesity and the associated comorbidities of excess weight are well documented and represent a major challenge to public health and the health care systems. [1]–[4] Regular exercise is considered a central component of weight loss and there are many organizations that recommend an hour or more per day of exercise to prevent weight gain, promote weight loss and/or prevent weight regain. [1], [5]–[8] Given the potential for exercise to create a negative energy balance, the relative importance of exercise in promoting weight loss is far more complicated than expected as exercise-based weight loss studies produce considerably less weight loss than predicted. For example, in a comprehensive review of clinical exercise and weight loss trials, Ross et al noted that in studies lasting more than 25 weeks in duration the average weight loss was only ∼30% of predicted. [9] The difference between actual weight loss and predicted weight loss has been termed “compensation” and participants whose exercise induced weight loss is less than predicted based on caloric expenditure have been identified as “compensators”. [10] In all previous exercise studies exploring weight compensation, only one dose of exercise was examined and these were typically large doses of exercise prescribed specifically for weight loss. [9]–[11]

This is important because the exercise recommendation for general health is 30 minutes per day on most days of the week, which is considerably less than the weight loss recommendation of 60 minutes per day most days of the week. [1], [5]–[8], [12], [13] To our knowledge there are no studies that have examined weight compensation across different doses of exercise. The Dose-Response to Exercise in postmenopausal Women (DREW) study was designed to examine the health benefits of 50%, 100%, and 150% of the NIH Consensus Panel physical activity recommendation in sedentary, overweight or obese, postmenopausal women with elevated blood pressure. [14], [15] The results from the primary outcomes, cardiorespiratory fitness and blood pressure, have been reported but due to the large undertaking and costs associated with conducting the DREW trial a number of important secondary outcomes were included *a priori* in the study design including changes in weight. [15] In DREW, participant retention was excellent (92%), non-exercise activity was monitored and the exercise training was supervised with outstanding adherence (∼97% for completers). [15] Thus the DREW study represents a unique opportunity to examine actual compared to predicted weight loss (compensation) across different doses of exercise.

## Methods

A complete description of the DREW design, methods and primary outcomes has been published elsewhere.[14], [15] In brief, the study was a randomized, dose-response exercise trial with a no-exercise control group and 3 exercise groups with incrementally higher doses of energy expenditure. The study was originally reviewed annually by The Cooper Institute and subsequently approved by the Pennington Biomedical Research Centers Institutional Review Board (IRB) for continued analysis. Prior to participation, all participants signed a written informed consent document outlining the procedures involved in the DREW study. The protocol for this trial and supporting CONSORT checklist ate available as supporting information; see Checklist S1 and Protocol S1.

**Figure 1:**
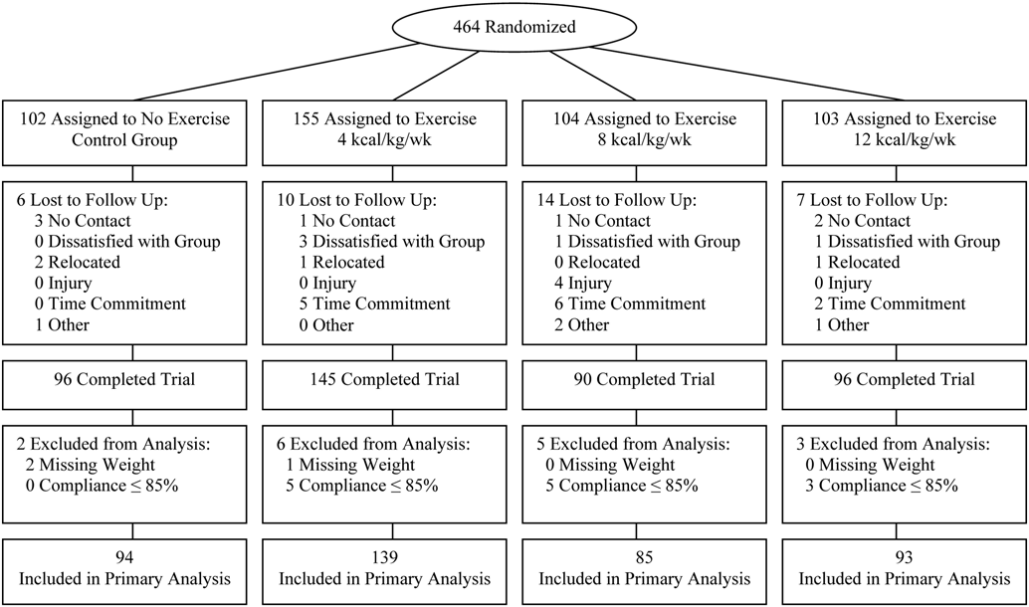
Participant flow diagram.

An abbreviated CONSORT diagram is provided in **Figure 1**. The full CONSORT diagram for the DREW study has been previously published. [15] We conducted a total of 4545 telephone screens between April 2001 and June 2005. After baseline testing, a two week run-in period, and giving informed consent, 464 postmenopausal women within the age range of 45 to 75 years, who were sedentary (not exercising more than 20 minutes on 3 or more day a week, and <8000 steps per day assessed over the course of one week), overweight or obese (BMI 25.0 to 43.0 kg/m^2^), and had a systolic blood pressure of 120.0 to 159.9 mm Hg were randomized to 1 of 3 exercise groups or a non-exercise control. Exclusion criteria included history of stroke, heart attack, or any serious medical condition that prevented participants from adhering to the protocol or exercising safely. Participants were recruited using a wide variety of techniques including newspaper, radio, television, mailers, community events, and email distributions. [14], [15]

The non-exercise control group was asked to maintain their baseline levels of activity and dietary habits during the six-month study period. All participants were asked to record their daily steps (see below) and fill out monthly medical symptoms questionnaire forms.

We calculated the prescribed exercise energy expenditure for women in the DREW study to meet the consensus public health recommendation from the NIH and other organizations.[13], [16] We used data from sedentary women recruited for previous exercise trials conducted by our group, and also from our large cohort study.[17]–[20] Specifically, we estimated that the typical sedentary postmenopausal woman who started an activity program and followed the consensus public health recommendation would expend 8 kcal/kg/week (KKW) in the exercise program. Details of these calculations are presented in the DREW design and methods report.[14] A major objective of DREW was to evaluate exercise levels 50% below and 50% above the current public health recommendations to test whether the lower dose provides any benefit and whether the higher dose provides proportionally more benefit than the standard 8-KKW exercise level. Thus, women were assigned to either a non-exercise control group or to groups that expended 4, 8, or 12 KKW. Exercising women participated in 3 or 4 training sessions each week for 6 months with training intensity at the heart rate associated with 50% of VO_2_. During the first week, each group expended 4 KKW. Those assigned to the 4 KKW group remained at this dose for the duration of the study while those assigned to the 8 and 12 KKW groups continued to increase their energy expenditure 1 KKW until their assigned exercise level was reached. All exercise sessions were performed under supervision in an exercise laboratory, and strict monitoring of the amount of exercise completed during each session was performed. Participants were weighed each week, and their weight was multiplied by their exercise dosage to determine the number of calories to be expended for the week. Women in the exercise groups alternated training sessions on semi-recumbent cycle ergometers and treadmills. Adherence to exercise training over the entire 6-month period was calculated for each individual by dividing the caloric expenditure during the exercise training by the caloric expenditure prescribed for the training period ×100%

Fitness testing was conducted using a Lode Excalibur Sport cycle ergometer (Groningen, Netherlands), an electronic, rate-independent ergometer. Participants cycled at 30 Watts (W) for 2 min, 50 W for 4 min, followed by increases of 20 W every 2 min until they could no longer maintain a pedal cadence of 50 rpm. Respiratory gases were measured using a Parvomedics True Max 2400 Metabolic Measurement Cart. Breath-by-breath respiratory gases were measured using a Parvomedics True Max 2400 Metabolic Measurement Cart. Volume and gas calibrations were conducted before each test. Gas-exchange variables (VO2, CO2 production, ventilation, and respiratory exchange ratio [RER]) were averaged every 15 s. Heart rate was measured directly from the ECG monitoring system. Ratings of perceived exertion (RPE) were obtained using the 20-point Borg scale. Two fitness tests were performed on separate days at baseline and follow-up, and the average value from these two tests were used for baseline and follow-up values for analyses.

There were two sources of weight data. The primary analysis was performed using the weights measured at baseline and follow-up in the clinical assessment laboratory. These visits occurred in the morning under fasting conditions. Weight was measured on an electronic scale (Siemens Medical Solutions, Malvern, PA) and height was measured using a standard stadiometer. This scale was calibrated weekly. Body mass index (BMI) was calculated as weight in kilograms divided by height in meters squared. For the individuals in the exercise groups there was a second source of weight data available for analysis. These were the weights taken during the first exercise session of each week in the exercise training room. We used the same type of electronic scale for these measurements and each scale was calibrated weekly. These weights were used to determine the exercise prescription for the week and were taken in the non-fasted state with participants wearing their workout clothes but no shoes. Waist and hip circumference were measured using the recommendations of the Airlie Conference.[21] Body fat was estimated from the sum of four skinfolds; triceps, abdomen, iliac crest and thigh. [22]

To calculate predicted weight change we assumed that 1 Kg of weight represented 7700 kcal in energy. Caloric expenditure from supervised exercise (total and weekly) was divided by 7700 kcal to predict weight change.

To assess potential changes in nonsupervised physical activity, all randomized participants wore a step counter (Accusplit Eagle, Japan) over the 6-month experimental trial to record their daily steps. Exercisers removed the step counter during supervised exercise sessions. Smoking history and medication use were assessed by detailed questionnaire. Diet was assessed by the Food Intake and Analysis System semi-quantitative food frequency questionnaire. [23] Participants were repeatedly informed that the study was not a weight loss trial and were asked not to change their dietary or physical activity habits.

The primary purpose of our current analysis was to examine weight compensation across different doses of exercise. Accordingly, we limited our analysis to participants with baseline and follow-up weight values and in participants randomized to an exercise group we excluded individuals with a supervised exercise energy expenditure adherence of 85% or less.

Descriptive baseline characteristics of groups were tabulated as means and SDs or as percentages, but not tested for differences. We calculated average step data per month for each randomization group. Compensation was defined as the difference between predicted and actual weight loss. Participants whose weight compensation was greater than zero were defined as compensators. For each group we also calculated the mean percent of predicted weight loss achieved (actual/predicted ×100%). Weight compensation cannot occur in the control group; therefore, their data were not eligible for analysis. Differences in outcomes among the randomization groups were tested by ANOVA with adjustment as appropriate. For statistically significant ANOVAs (p<.05), all pairwise comparisons among the randomization groups were tested using Tukey studentized range adjustment. Results are presented as adjusted least-squares means with confidence intervals. For each exercise group, we examined mean predicted weight loss, actual weight loss and compensation for weeks 1 through 23. Using ANOVAs (p<.05) with adjustment for multiple comparisons we examined if the mean compensation for each group was statistically different from zero for each week of the intervention. This analysis was limited to individuals with at least 19 weeks with recorded body weight data. Body weight values from the previous week were carried forward when body weight data was missing.

All reported *P* values are two-sided. All analyses were performed using SAS version 9.0 (Cary, NC).

## Results

Of the 464 randomized participants, 427 returned for follow-up testing of which 3 individuals had missing weight data and 13 of the participants from exercise groups had an adherence value ≤85%, resulting in 411 (88.6%) participants with complete data. **Table 1** summarizes the participant descriptive characteristics by group. The study sample had a mean age of 57.2 (6.3), BMI of 31.7 (3.8) and 63.5% of the sample was Caucasian. The mean adherence to the exercise prescription was 99.5, 99.3 and 99.2% across the 4, 8 and 12 KKW groups, respectively. To assess potential changes in non-exercise activity daily step counts were examined for individuals with complete step counter data. The mean steps per day at baseline for all participants were 4858 (1715) with no significant differences between groups. The mean steps per day during the first month of intervention were 5399 (1873), 5288 (1870) and 5264 (1933) for the 4, 8 and 12 KKW group respectively. There were no significant differences in mean weekly steps during the first month among any of the exercise intervention groups, but the non-exercise control group (6098 (2065)) had significantly (P<0.05 for each) more steps compared to all of the exercise groups. However, by the 6^th^ month of intervention there was no statistically significant difference in mean steps between any of the groups. During month 6 the daily steps for the control group was 5830 (2046) and 5416 (2054), 5444 (1866) and 5473 (1749 for the 4, 8 and 12 KKW groups. respectively. This finding is virtually identical to what we observed with the full cohort. [15] Also similar to previous reports from the full study cohort there was a dose response in change in fitness (P-trend<0.001) across the study groups with the 4, 8 and 12 KKW groups having a 3.7 (95% CI:1.9, 5.4), 6.9 (4.7, 9.2), and 8.0 (5.9, 10.2) percent change in fitness, respectively, each of which was statistically significant (p<0.001) compared to the control group (−2.7 (−4.9, −0.6)). [15]

**Table 1:** Participant Characteristics*.

Characteristics		Randomization Groups			
	All	Control	4 kcal/kg/wk	8 kcal/kg/wk	12 kcal/kg/wk
	(n = 411)	(n = 94)	(n = 139)	(n = 85)	(n = 93)
**Demographics**					
Age, y	57.2 (6.3)	57.2 (5.9)	57.9 (6.5)	56.7 (6.4)	56.4 (6.3)
Ethnicity, No. (%)					
Caucasian	261 (63.5)	62 (65.9)	83 (59.7)	49 (57.7)	67 (72.0)
African American	123 (29.9)	23 (24.5)	47 (33.8)	29 (34.1)	24 (25.8)
Hispanic/other	27 (6.6)	9 (9.6)	9 (6.5)	7 (8.2)	2 (2.2)
Weight, kg	84.2 (11.9)	85.6 (12.4)	83.4 (11.6)	85.0 (12.8)	83.1 (11.1)
Body mass index, kg/m^2^ †	31.7 (3.8)	32.2 (3.9)	31.4 (3.7)	32.2 (4.1)	31.1 (3.6)
Body fat, %	28.9 (4.9)	30.8 (5.5)	27.6 (4.1)	29.3 (4.8)	28.7 (4.8)
Waist circumference, cm	100.9 (11.8)	102.8 (12.0)	100.0 (11.3)	101.9 (12.0)	99.2 (11.9)
Peak absolute VO_2_, L/min	1.30 (0.24)	1.33 (0.27)	1.28 (0.24)	1.28 (0.22)	1.32 (0.23)
Peak relative VO_2_, ml/kg/min	15.6 (2.8)	15.7 (2.9)	15.5 (2.9)	15.1 (2.2)	16.1 (2.8)
**Data From Exercise Training Laboratory**					
Total Exercise Energy Expenditure Prescribed, Kcal	N/A	N/A	7975 (1188)	15305 (2235)	20730 (2753)
Total Exercise Energy Expenditure Achieved, Kcal	N/A	N/A	7932 (1193)	15197 (2249)	20560 (2734)
6-Month Adherence, %	N/A	N/A	99.5 (2.1)	99.3 (1.8)	99.2 (2.7)
Time, min/week	N/A	N/A	72.2 (12.4)	136.3 (19.4)	193.7 (31.0)

Abbreviations: VO_2_, volume of oxygen consumed, N/A, not applicable.

* Continuous variables presented as means (SD) and dichotomous variables presented as count (percentage).

† Calculated as weight in kilograms divided by height in meters squared.

**Figure 2:**
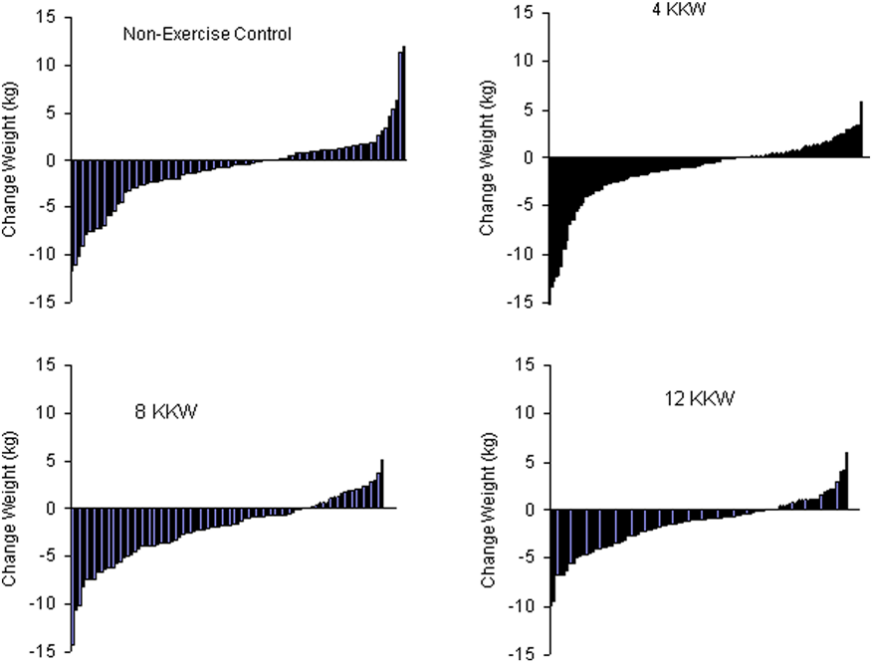
Distribution of weight loss for each study group.

**Figure 3:**
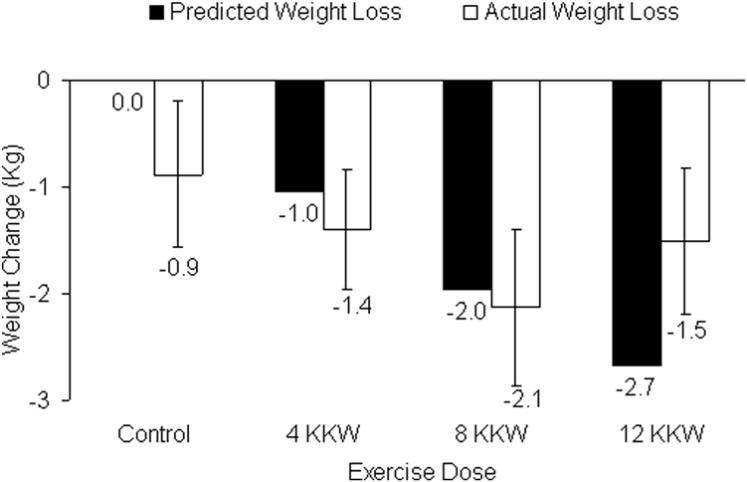
Actual weight loss (white bars) and predicted weight loss (black bars) for each study group. Actual weight loss was adjusted for age, baseline weight and ethnicity. Predicted weight loss represents caloric expenditure from supervised exercise divided by 7700 kcal/kg. Error bars represent 95 percent confidence intervals.

**Figure 2** depicts the heterogeneity in the individual amount of weight change in each of the groups. As depicted in **Figure 3**, all groups had a significant reduction in weight compared to weight at baseline (P<0.05 for each) but there were no significant differences between groups in weight change (P>0.05 for all between group comparisons). Change in weight was adjusted for age, baseline weight and ethnicity. Within the exercise groups the predicted weight change in the 4 and 8 KKW groups closely matched the actual weight loss while in the 12 KKW group the actual weight loss (−1.5 kg) was considerably less than the predicted weight loss (−2.7 kg). Analyses repeated without adjustment produced similar results.

**Figure 4:**
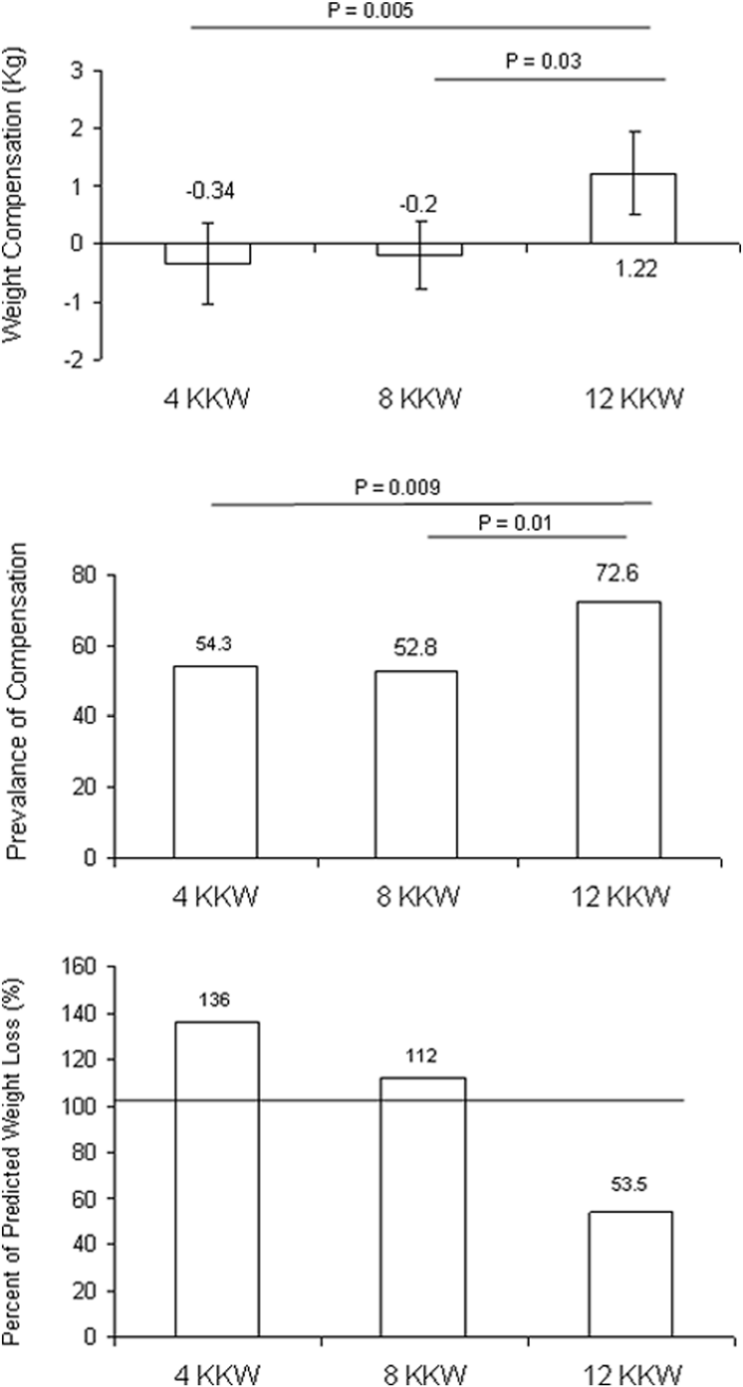
The top panel depicts weight compensation (actual weight loss minus predicted weight loss) across exercise groups. The middle panel depicts the prevalence of compensation across exercise groups and the lower panel presents the percent of predicted weight loss for each exercise group. All values adjusted for age and ethnicity. Error bars in the top panel represent 95 percent confidence intervals.

The three panels of **Figure 4** more closely depict compensation with all values adjusted for age and ethnicity. In the top panel, the amount of compensation in each exercise group is plotted. The 12 KKW (1.2 kg) had significantly greater compensation compared to the 4 and 8 KKW groups (−0.3 and −0.2 kg, respectively). The middle panel depicts the prevalence of compensation in the exercise groups. In the 4 and 8 KKW groups, 54.3% (p = 0.009) and 52.8% (p = 0.01) of participants, respectively, were compensators both of which were lower compared to 72.6% in the 12 KKW group. The lower panel summarizes the percent of predicted weight loss achieved in each of the exercise groups. The 4 and 8 KKW achieved over 100% of the predicted weight loss while the 12 KKW group only achieved 53.5% of the predicted weight loss.

**Figure 5:**
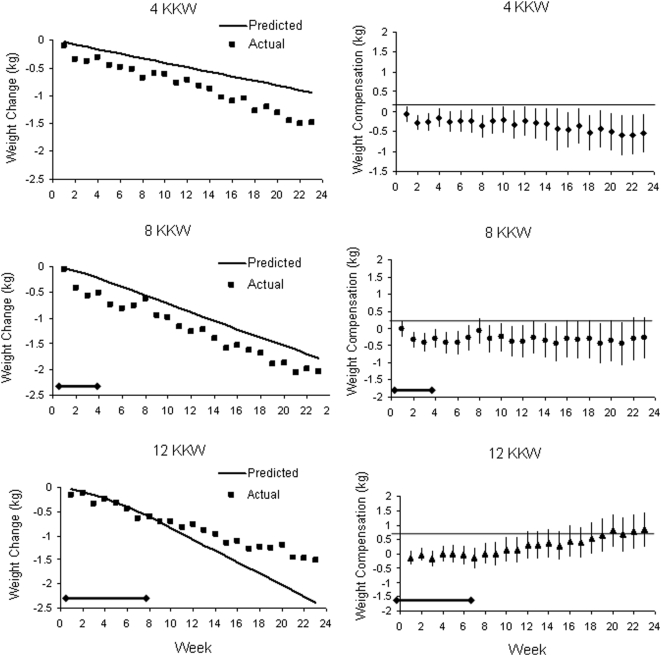
Predicted weight loss and actual weight loss are presented in the left column while compensation is presented in the right column for each exercise group during weeks 1 through 23 of the intervention period. This analysis was limited to individuals with at least 19 weeks with recorded weights and for missing values the weight from the previous week was carried forward. This resulted in 132, 84 and 87 participants used in the analysis for the 4, 8 and 12 KKW groups, respectively. It should be noted that exercise during week's 1–4 for the 8 KKW group and weeks 1–8 for the 12 KKW group were not performed at the full dose as these weeks were part of the exercise dose ramping phase. For compensation, statistical deviation from zero for each week was tested using ANOVAs with adjustment for multiple comparisons.

**Figure 5** graphically depicts body weight data obtained in the exercise training lab for determination of exercise prescription. It should also be reiterated that this analysis was limited to individuals with at least 19 weeks with recorded weights resulting in 132, 84 and 87 participants used in the analysis for the 4, 8 and 12 KKW groups, respectively. The left column of the graphs compares the actual weight loss to predicted weight loss for each exercise group during weeks 1 through 23 of the intervention period. The right column of graphs plots the compensation during the exercise intervention for each of the groups. For the 4 KKW group, not only was there no compensation but the weight loss was more than expected at weeks 2, 3, 8, 16 and 18 through 23. For the 8 KKW group the weight loss was more than expected at weeks 2 through 6; however during weeks 7 through 23 actual and predicted weight loss were closely matched. For the 12 KKW group there was no evidence of compensation until week 10, at which point compensation started to trend upward and reached significance at week 19 and remained significant for the remainder of the intervention weeks. It should be noted that exercise during weeks 1–4 for the 8 KKW group and weeks 1–8 for the 12 KKW group were not performed at the full dose, as these weeks were part of the exercise dose ramping phase.

**Table 2** summarizes the energy intake data from baseline and follow-up across groups. Both at baseline and follow-up there were no between group differences in mean energy intake. However, for all groups, the reported mean energy intake was lower at follow-up compared to baseline. There were no between groups difference in baseline or follow-up in dietary protein, fat and carbohydrate intake (data not included). To explore mechanisms of weight loss in the control group, we categorized individuals in the control group into those that lost weight (n = 51) and those that maintained or increased weight (n = 37), and we examined mean follow-up energy intake adjusted for baseline energy intake. The individuals in the control group that lost weight had a lower energy intake at follow-up compared to individuals in the control group that did not lose weight (1766 [1618, 1927] versus 2035 [1820, 2239] kcal, P = 0.04). In the exercise groups we observed no difference in mean follow-up energy intake adjusted for baseline energy intake between the compensators and non-compensators. Given the considerable limitations of our self-report food frequency questionnaire, all the dietary results need to be interpreted with caution.

**Table 2:** Energy Intake at Baseline and Follow-up.

	Randomization Groups				
	Control	4 KKW	8 KKW	12 KKW	Between Groups P-Value
	(n = 88)	(n = 120)	(n = 77)	(n = 80)	
Energy Intake, kcal/day*					
Baseline	2138 (1995, 2344)	2000 (1866, 2138)	2228 (2040, 2399)	2188 (2040, 2399)	.16
Follow-Up	1862 (1698, 1995)†	1730 (1585, 1819)†	1831 (1656, 1995)†	1870 (1698, 2040)†	.42

Abbreviations: KKW, kcal/kg/week.

* Values are expressed as fitted mean (95% CI) of normalized data.

† Within Group Differences Between Baseline and Follow-Up P<0.001.

**Figure 6:**
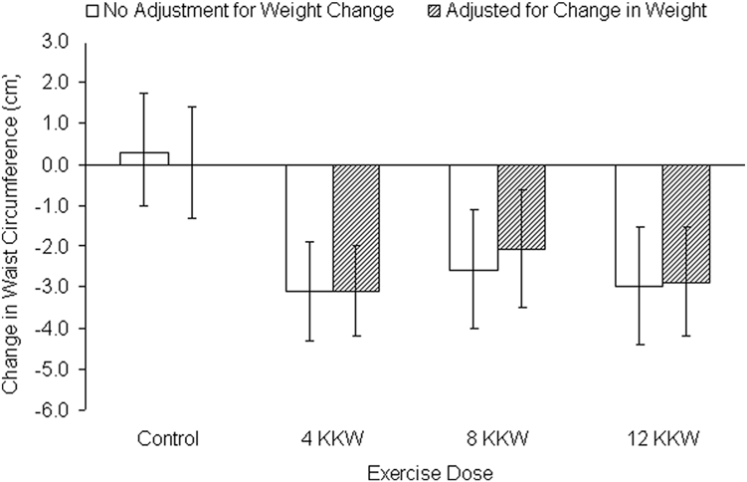
Change in waist circumference for each group with (diagonal striped) and without (open) adjustment for change in weight. The change in waist circumference was significantly different from control for each exercise groups in both analyses. All values adjusted for age and ethnicity. Error bars in the top panel represent 95 percent confidence intervals.

**Figure 7:**
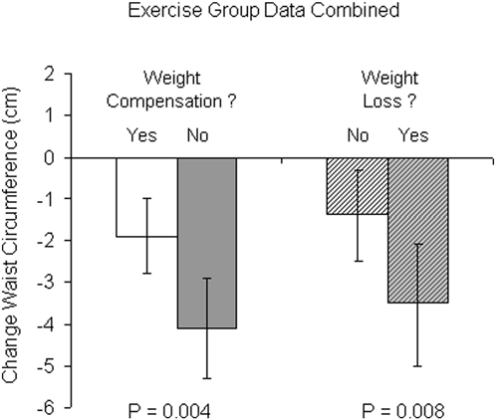
Changes in waist circumference within stratifications of weight compensation and weight loss with the all data from the exercise groups combined. The set of bars to the left represent change in waist circumference with categorization by weight compensation (yes/no) and set of bars to the right represent categorization by weight loss (yes/no). All values adjusted for age and ethnicity. Error bars in the top panel represent 95 percent confidence intervals.

Because changes in weight do not provide any insight into where the weight has changed or whether the weight loss is primarily fat, muscle or both we examined changes in body fat and waist circumference. The change in body fat was not statistically different across the control, 4,8 and 12 KKW groups: 1.0 (−0.1, 2.1), −0.7 (−1.6, 0.2), −0.5 (−1.7, 0.6) and −0.1 (−1.2, 1.0), respectively (p>0.05 for each). In contrast, as depicted in Figure 6 all exercise groups had a reduction in waist circumference compared with the control group and these decreases were largely unchanged by adjustment for changes in weight. Interestingly the correlation between change in weight and change in waist circumference was only 0.34 (p<0.001) ), indicating that weight change accounted for only 11.6% of the variance in waist circumference change. To further explore the exercise-weight loss and waist circumference relations, we examined changes in waist circumference within stratifications of weight compensation and weight loss with the all data from the exercise groups combined. As depicted in Figure 7, with both the weight compensators and non-compensators there were significant decreases in waist circumference but reduction in waist circumference in the non-compensators was approximately twice as much as observed in the compensators. Similar results were found when the combined exercise data was stratified by weight loss (yes/no). These data suggest that waist circumference is decreased in response to exercise training even in the absence of weight loss but that when weight loss is present the decrease in waist circumference is significantly greater.

## Discussion

The primary finding from this large exercise intervention trial in postmenopausal women is that the difference between actual weight loss and predicted weight loss (compensation) increases with exercise dose. We confirmed the findings of previous studies that a relatively high dose of exercise (12 KKW or 194 minutes per week) results in compensatory mechanisms that attenuate weight loss in previously sedentary women. However, a dose of exercise consistent with the exercise prescription for general health (8 KKW or 136 minutes per week) did not result in compensation as the actual weight loss closely matched the predicted weight loss. An exercise dose of 4 KKW (72 minutes per week) also resulted in weight loss that slightly exceeded the predicted weight loss. Our findings are important because most exercise guidelines for weight loss recommend 200–300 minutes per week and we provide evidence that this amount of exercise induces compensation that results in significantly less weight loss than predicted. Our findings of different doses of exercise resulting in different amounts of compensation may in part explain the discrepant results from previous exercise and weight loss studies. If different doses of exercise result in different amounts of compensation it is not surprising that when exercise studies of varying doses and duration are examined collectively there is no relation between exercise dose and weight loss. Our findings provide an excellent example of the complex nature of the energy expenditure and energy intake relation, and the importance of considering both sides of the equation in creating weight loss programs.

Numerous well conducted clinical exercise trials of long duration have reported less weight loss than expected. [9] For example, Donnelly et al observed that after 18 months of exercise training and achieving 2000 kcal per week of exercise, college-aged women had no weight loss, although women in the control group actually gained weight. [11] In a follow-up report Donnelly et al suggested that the observed “compensation” was due to increased energy intake, not changes in metabolic rate or non-exercise activity. [24] A recent report by King et al provides support that increased energy intake may be a major source of energy compensation as they noted that after 12 weeks of 2500 kcal per week in exercise training energy expenditure, in those individuals that did not lose the predicted amount of weight (compensators) there was a increase in caloric intake while in those that lost the predicted amount of weight (non-compensators) there was no change in caloric intake. [10] Unlikely sources of the observed compensation in the DREW study include changes in resting metabolic rate and the thermogenic effect of food as previous studies have found these variables not to be influenced by exercise training. [24] Our step counter data suggests that there was no change in outside activity in the exercise groups, nor were there any differences in average daily steps among the exercise groups. [15] Though doubly-labeled water or accelerometer data would have presumably been more accurate than our step data, it is still unlikely that substantial changes in non-exercise activity explain our findings.

In the previously published DREW primary outcomes paper we reported that the mean follow-up waist circumference was lower in all exercise groups compared to the control group and there was no difference in mean follow-up weight across groups. [15] The current analysis allowed us to further explore the change in weight data including examining individual level data as well as weight compensation. Further we were able to examine if exercise induced changes in waist circumference are independent of weight loss. Our observation that adjusting for changes in weight did not meaningfully affect the change in waist circumference in any of the exercise groups as well as the observation that even in the exercise sub-group that did not lose weight there was a reduction in waist circumference is in agreement to previous published reports. [25] It is worth noting that the exercisers that did lose weight had approximately two times the loss of waist circumference compared to the exercisers that did not lose weight which is also in agreement with previous reports. [25] Excess abdominal obesity is associated with increased risk of mortality, CVD, diabetes, insulin resistance and metabolic syndrome. [26]–[29] These findings reinforce the positive health benefits of engaging in physical activity even in the absence of substantial weight loss.

The DREW study was not designed to examine differences in actual and predicted weight loss nor the mechanism responsible for these observations. As such our measures of food intake, a food frequency questionnaire, is not optimal to measure small changes in energy intake. While we cannot conclude that an increase in energy intake in response to increased energy expenditure is the source of the observed compensation, based on the work of others we conclude it is the most likely cause.

Our findings should not be interpreted as suggesting that lower doses of exercise are more effective in producing weight loss than higher doses. We emphasize that DREW was not a weight loss study and it was not designed to examine the nuances of exercise induced weight loss. As such we hope our findings stimulate more research into the variability and mechanisms of exercise induced compensation. In the 12 KKW group approximately 27% of individuals did not have significant compensation in response to exercise training suggesting there are individuals that can achieve higher doses of exercise without compensation. Though primarily focused on weight loss maintenance, not weight loss, data from the Weight Control Registry (WCR) demonstrates that individuals who have lost a substantial amount of weight and have maintained weight loss typically achieve 45 or more minutes a day of moderate intensity activity. [30] These data from the WCR provides further data that there are individuals that either are not prone to compensation or who have developed strategies to combat compensation. Developing methods to identify individuals likely to compensate, and developing/testing strategies to combat this compensation are areas deserving of future work.

Our findings also offer important insight into the exercise dose required for the prevention of weight gain. Based largely on data from cross-sectional doubled labeled water studies, the 2005 USDA Dietary Guidelines and Institute of Medicine recommend obtaining at least 60 minutes of moderate intensity activity per day to prevent weight gain. [6], [8] Both the 4 and 8 KKW group in DREW not only did not gain weight but actually lost weight during the 6 month study period. These two groups performed 72 and 136 minutes per week of moderate intensity physical activity, which is considerably less than the recommended 60 minutes per day to prevent weight gain. Our findings highlight the need for more research, in particular large randomized controlled clinical trials, exploring the dose of physical activity required to prevent weight gain as 60 minutes a day may present itself as a daunting undertaking for most individuals but even more so for a habitually sedentary individuals. In addition, this dose may well lead to compensation by more individuals, and to less success in weight loss.

Strengths of the DREW study include that it is an efficacy study, using a tightly controlled exercise doses, with all exercise completed in the laboratory and extensive monitoring of exercise energy expenditure. We obtained excellent exercise adherence and had a low dropout rate. Monitoring of steps per day throughout the 6-month period indicates that outside physical activity remained constant throughout the trial for all exercise groups, thus ensuring that observed group differences were due to the prescribed exercise dose. Further, the exercise doses, including intensity, are easily obtainable and are well tolerated by sedentary women; and this has important public health implications for refining future physical activity recommendations.

The study has limitations because the sample is limited to sedentary, overweight or obese, postmenopausal women with elevated blood pressure. Thus, we do not know if the results will apply to other women or men. However, the study sample is a group that is likely to benefit from exercise training and represents a sizeable proportion, probably a majority, of U.S. women in the age range of 45 to 75 years. Further the study could have benefitted from a more rigorous and sensitive measure of energy intake. The food frequency questionnaire utilized in DREW prevented us from being able to definitively demonstrate that the observed compensation is the result of an increase in energy intake. However, it should be reiterated that examining compensation was not the *a priori* goal of DREW. The exercise training intensity was moderate and while this makes for good public health and clinical applicability, it is possible that higher levels of training intensity might produce different results in regard to compensation and changes in waist circumference.

In this study of previously sedentary, overweight or obese, postmenopausal women we observed no difference in the actual and predicted weight loss with 4 and 8 KKW of exercise (72 and 136 minutes respectively), while the 12 KKW (194 minutes) produced only about half of the predicted weight loss. We need to gain a better understanding of the mechanisms responsible for this exercise dose dependent phenomenon and develop strategies to identify and treat potential compensators.

## Supporting Information

Checklist S1 CONSORT Checklist
(0.13 MB PDF)

Protocol S1 Trial Protocol
(0.47 MB PDF)
