# Supplementary material for: Changes in Weight, Waist Circumference and Compensatory Responses with Different Doses of Exercise among Sedentary, Overweight Postmenopausal Women
Source: PLoS One. 2009 Feb 18;4(2):e4515. doi: 10.1371/journal.pone.0004515 (PMC2639700; doi:10.1371/journal.pone.0004515)
Supplement: Protocol S1 — Trial Protocol (0.47 MB PDF) [file pone.0004515.s002.pdf]

SPECIAL COMMUNICATIONS

Methodological Advances

Dose-Response to Exercise in Women Aged 45–75 yr (DREW): Design and Rationale

GINA M. MORSS[1], ALEX N. JORDAN[1], JAMES S. SKINNER[2], ANDREA L. DUNN[1], TIMOTHY S. CHURCH[1], CONRAD P. EARNEST[1], JAMES B. KAMPERT[1], RADIM JURCA[1], and STEVEN N. BLAIR[1]

[1]*Centers for Integrated Health Research, The Cooper Institute, Dallas, TX; and* [2]*Department of Kinesiology, Indiana University, Bloomington, IN*

ABSTRACT

MORSS, G. M., A. N. JORDAN, J. S. SKINNER, A. L. DUNN, T. S. CHURCH, C. P. EARNEST, J. B. KAMPERT, R. JURCA, and S. N. BLAIR. Dose-Response to Exercise in Women Aged 45–75 yr (DREW): Design and Rationale. *Med. Sci. Sports Exerc.*, Vol. 36, No. 2, pp. 336–344, 2004. **Introduction and Purpose:** Physical inactivity in postmenopausal women contributes to a rise in atherogenic risk factors associated with the metabolic syndrome. Although regular physical activity positively contributes to health, inactivity progressively increases with age. The Dose Response to Exercise in Women aged 45–75 yr (DREW) study is designed to investigate the effect of different amounts of exercise training on cardiorespiratory fitness and risk factors for cardiovascular disease (CVD) in postmenopausal women at moderately increased risk of CVD. **Methods:** DREW will recruit 450 sedentary, healthy, postmenopausal women with a body mass index of 25–40 $kg \cdot m^{-2}$, resting systolic blood pressure (BP) of 120–159 mm Hg, and a resting diastolic BP of ≤ 99 mm Hg. Laboratory and self-report measures completed at baseline and 6 months include maximal oxygen consumption ($\dot{V}O_{2max}$), resting BP, anthropometry, dietary habits, physical activity history, medication use, menstrual history, personal and family medical history, and fasting HDL cholesterol, LDL cholesterol, triglycerides, and glucose. Eligible participants are randomly assigned to a nonexercise group or one of three exercise groups. Participants exercise 3 to 4× wk^{-1} at a heart rate equivalent to 50% of $\dot{V}O_{2max}$ expending 4, 8, or 12 $kcal \cdot kg^{-1} \cdot wk^{-1}$, depending on group assignment. This study will allow quantification of possible dose-response relations (50%, 100%, and 150% of the consensus physical activity recommendation) between exercise training and study outcomes. **Conclusion:** DREW can make important contributions to our understanding of the effects of physical activity in postmenopausal women and help refine public health and clinical recommendations for this group. **Key Words:** AEROBIC TRAINING, BLOOD PRESSURE, FITNESS, PHYSICAL ACTIVITY, INTENSITY

About 38% of American women (over 25 million) are postmenopausal. Because of an increasing life expectancy, women generally spend one-third of their lives in this phase of the lifespan (28). Menopause is associated with a rise in atherogenic risk factors, e.g., 1) an increase in blood pressure (BP) (especially systolic BP [SBP]); 2) a worsening of the blood lipid profile; 3) a progressive gain in body weight, with about 66.1% of women overweight and 37.8% obese by age 40–59 (12); and 4) an increased tendency for central obesity with excess intra-abdominal visceral fat deposition, hyperinsulinemia, and the associated metabolic syndrome. Physical inactivity in postmenopausal women contributes to the increase in these risk factors and to low cardiorespiratory fitness (8,15). About 30% of women in the postmenopausal age range report no physical activity at all, and the prevalence of inactivity progressively increases with age (30). Further, African-American women are more likely than Caucasian women to be sedentary (30).

Regular physical activity makes positive contributions to health and well-being (14,23,24,30). The consensus recommended dose described in guidelines is perhaps most clearly presented as "obtaining 30 min of moderate-intensity physical activity, such as brisk walking, on most, preferably all, days of the week." Despite general agreement that 150–210 min weekly of moderate-intensity physical activity reduces mortality and cardiovascular disease (CVD) risk, there are still uncertainties about the specific types, amounts, patterns, and intensities of exercise that produce optimal or even measurable effects. Although there is agreement in current activity guidelines on a general dose-response relationship between exercise and health benefits, data to quantify this relationship are not compelling. Unanswered ques-

Address for correspondence: Gina M. Morss, The Cooper Institute, 12330 Preston Road, Dallas TX 75230; E-mail: gmorss@cooperinst.org.
Submitted for publication May 2003.
Accepted for publication October 2003.

0195-9131/04/3602-0336
MEDICINE & SCIENCE IN SPORTS & EXERCISE®
Copyright © 2004 by the American College of Sports Medicine
DOI: 10.1249/01.MSS.0000113738.06267.E5

Copyright © American College of Sports Medicine. Unauthorized reproduction of this article is prohibited.

tions with important practical and clinical implications include:

- "Will sedentary individuals get any health benefit if they perform less physical activity than the recommended amount?"
- "If individuals perform more physical activity than the recommended amount, will they obtain greater (or proportionally greater) health benefits?"
- "Does the dose-response relationship between exercise and outcomes vary for different outcome measures?"

After reviewing evidence from exercise training studies on the inverse relationship between physical activity and mortality, we conclude that total energy expenditure is the most important component of an exercise program for health promotion (5,6). If this is correct, the implication is that health-related outcomes such as CVD risk factors and cardiorespiratory fitness can be improved with various combinations of frequency, duration, and intensity of exercise. A question remains concerning whether there are "minimal," "threshold," or "optimal" doses of exercise needed to improve fitness and health. The Dose-Response to Exercise in Women aged 45–75 yr (DREW) study is designed to provide information on this and other related questions. Funded by the National Heart, Lung, and Blood Institute, the DREW study is designed to investigate the effect of different amounts of exercise training on cardiorespiratory fitness and risk factors for CVD in sedentary, overweight, or obese postmenopausal women at moderately increased risk of CVD.

SPECIFIC OBJECTIVES

We will randomly assign sedentary postmenopausal women to one of three exercise groups or a nonexercise control group. Exercising women will participate in three or four training sessions each week for 6 months with energy expenditures of 4, 8, or 12 $kcal \cdot kg^{-1} \cdot wk^{-1}$ (KKW). Exercise intensity will be 50% of maximal oxygen consumption ($\dot{V}O_{2max}$). The objective is to precisely quantify the dose-response changes in the primary outcomes of $\dot{V}O_{2max}$ and resting SBP. Secondary outcomes include blood lipids and lipoproteins, body weight and composition, BP response to exercise, myocardial oxygen demand (estimated by the product of heart rate [HR] and SBP), fasting glucose, HR variability, quality of life and other psychosocial variables, and change in CVD risk as defined by a multiple logistic risk function (11). This study also will yield insights into the extent to which observed dose-response relationships are modified by baseline levels of fitness, ethnicity, risk factors, or age.

METHODS

Description and selection criteria of participants. There are few large, well-controlled studies on the effects of specific doses of exercise in postmenopausal women. DREW will recruit 450 sedentary, healthy, postmenopausal women aged 45 to 75 yr who are overweight or obese (body mass index [BMI] of 25–40 $kg \cdot m^{-2}$) and have normal or mildly elevated BP (resting SBP of 120–159 mm Hg and a resting diastolic blood pressure [DBP] of $\leq$ 99 mm Hg). These criteria are representative of a large number of postmenopausal women with a moderately elevated risk for CVD and who may benefit from exercise training. However, their risk is not so great that pharmacological intervention is initially required. According to established guidelines for treatment of elevated BP, lipids, and BMI, a 6-month lifestyle intervention is recommended (11,21,22). Approximately 65% of the participants will come from non-Hispanic white ancestry, and the remaining 35% will come from minority groups, primarily African-American and Mexican-American women. To enhance the recruitment of minorities, we have opened an offsite facility. Our facility occupies space in a medical clinic with a high concentration of minority women as patients. In addition, the community where the medical clinic is located is comprised primarily of minorities. Orientations, run-in sessions, and supervised exercise sessions are conducted at the facility for the convenience of the participants. However, the baseline and 6-month follow-up medical assessments are conducted at our campus in North Dallas.

In the DREW study, one or more of the following determines postmenopausal status:

- >55 yr old and no natural menses for at least 1 yr (the median age of natural menopause in North America is 51 yr, with a usual range of 48 to 55 yr).
- <55 yr old and no natural menses for at least 1 yr or taking hormone replacement therapy (HRT) for 1 yr.
- Aged 45–55 yr with medical documentation of a hysterectomy with a bilateral oophorectomy.
- <55 yr old with a hysterectomy but no documentation of a bilateral oophorectomy, taking HRT for 1 yr.

The inclusion and exclusion criteria for DREW are listed in Table 1. Women taking medication for thyroid, cholesterol, and BP problems are eligible as long as they have been on a stable dose for at least 3 months. Women taking medication with a beta-blocker are not eligible to participate because of the effect beta-blockers have on heart rate. HRT is allowed, as long as participants have been on a stable dose for a minimum of 6 months.

Timeline and rationale. Participant flow from screening through randomization is depicted in Figure 1. Initial eligibility is determined by a telephone interview to obtain information about age, physical activity, menstrual history, height, weight, current medical history and medication use, and residence. Individuals who remain eligible after the phone screen and express willingness to learn more about the study are invited to an orientation session. At the orientation session, potential participants are weighed and height measured to calculate BMI. A thorough explanation of the study is given, study requirements of the participants are explained, what participants can expect from the study is clarified, and any questions are answered. Eligible participants who wish to participate in further screening sign an

Copyright © American College of Sports Medicine. Unauthorized reproduction of this article is prohibited.

TABLE 1. Inclusion and exclusion criteria for DREW.

**Inclusion criteria**	
Physically inactive	Women must be sedentary and have an energy expenditure of <35 kcal·kg^{-1}·d^{-1} (6).
Postmenopausal	• >55 yr old and no natural menses for at least 1 yr<br>• <55 yr old and no natural menses for 1^{+} yr or using HRT for 1^{+} yr<br>• Aged 45–75 yr with medical documentation of a hysterectomy with a bilateral oophorectomy<br>• <55 yr old with a hysterectomy but no documentation of bilateral oophorectomy, using HRT for 1^{+} yr
Hormonal replacement therapy	HRT is allowed, if they have been on a stable dose for ≥6 months
BMI	25–40 kg·m^{-2}; height and weight measured and BMI calculated at the orientation session
Blood pressure	120–159 mm Hg systolic and ≤99 mm Hg diastolic; screening blood pressure measurements performed during the behavioral intervention/run-in<br>Women on a stable dose of antihypertensive medications during the past 3 months will be accepted, except women using beta-blockers
Physically capable of exercise	There is no minimal or maximal value for $\dot{V}O_{2\ max}$; participants must be able to exercise safely at the required doses
Informed consent	Women must be capable and willing to give written informed consent, understand exclusion criteria, and accept the randomized group assignment
**Exclusion criteria**<br>**A past history and/or physical examination or laboratory findings of the following medical conditions:**	
Significant cardiovascular disease or disorders	Including but not limited to arrythmias, myocarditis, cardiomyopathy, congestive heart failure, heart disease, stroke or transient ischemic cerebral attacks, peripheral vascular disease with intermittent claudication, acute, chronic, or recurrent thrombophlebitis
LDL cholesterol	LDL ≥130 mg·dL^{-1} and 10-yr risk >10% as indicated by NCEP Panel III report
Large weight loss	20 or more kg in the past year
Other exclusions	Hospitalization for mental illness within the past 5 yr or score ≥10 on the Center for Epidemiological Studies Depression scale (CESD)<br>Plans to be out of the city more than 4 wk over the next 6 months
Other significant medical conditions	Including but not limited to chronic or recurrent respiratory, gastrointestinal, neuromuscular, neurological, or psychiatric conditions; musculoskeletal problems interfering with exercise; autoimmune or collagen vascular diseases; immunodeficiency diseases or a positive HIV test; anemias, bleeding disorders, chronic thrombotic disorders, or hypercoagulable states; malignancies in the past 5 yr, with the exception of skin cancer therapeutically controlled; endocrine and metabolic disorders, including diabetes; any other medical condition or disease that is life-threatening or that can interfere with or be aggravated by exercise

informed consent approved by the Institutional Review Board of The Cooper Institute. Participants are then scheduled for their first run-in visit. Women who complete the run-in will become accustomed to working with our staff, resolve scheduling conflicts, and gain confidence in being able to make the time commitment for the study. A run-in is most useful when 1) poor adherence is associated with a substantially reduced "response" to training, 2) participants excluded by the run-in would have been unlikely to comply during the trial, and 3) the cost of the run-in and screening is low relative to training costs. In DREW, these conditions are clearly satisfied.

During the run-in period, participants visit The Cooper Institute six times over 2 wk to view a computer-based slide and audio intervention consisting of consensus recommendations from various guidelines on health-related topics (11,21,22,29). Topics include limiting alcohol intake, increasing physical activity, the importance of not smoking, and reducing the intake of sodium, saturated fat, and cholesterol. The importance of making these changes is emphasized, questions are answered, and appropriate educational materials from the National Heart, Lung, and Blood Institute, American Heart Association, American College of Sports Medicine, and other sources are distributed. This instruction in lifestyle modification exceeds what is typically given in clinical practice to women in these risk strata. Although the lifestyle intervention, especially the general exercise advice, might contribute to drift between treatment groups, a recent meta-analysis showed limited effects of short-term interventions such as ours (20). Delivering the intervention before randomization should not affect internal validity because any effect will be equally distributed across the groups. According to the study protocol for run-in, four to six BP measurements are taken during the first three run-in intervention sessions. After 5 min of rest, the first BP is measured. Consecutive measurements are taken every 2 min. The average BP is calculated for each session, with the

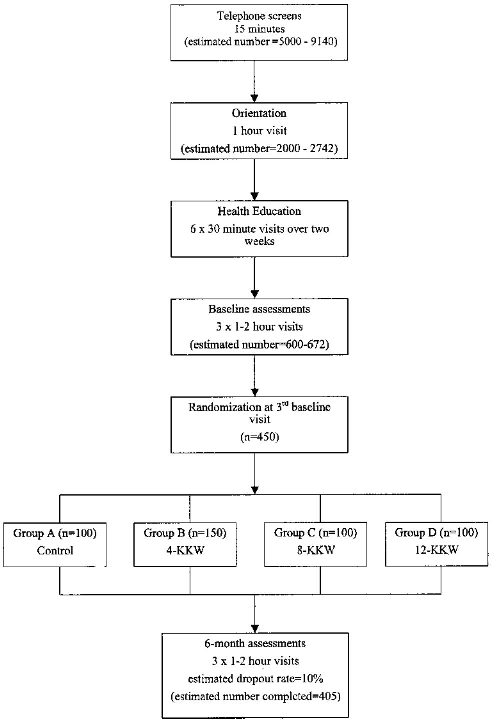

FIGURE 1—Study flow, participant time commitment for each level of the study, and the estimated number of participants screened at each level of the study.

Copyright © American College of Sports Medicine. Unauthorized reproduction of this article is prohibited.

first measure discarded. The average BP of the first three sessions is used to evaluate eligibility based on the BP criteria. Eligible participants complete the final three intervention sessions for continued adherence screening. Screening BP is measured manually with mercury manometers using the American Heart Association protocol (25).

Upon completion of the run-in, participants undergo baseline medical testing during which they are screened for the remaining inclusion/exclusion criteria such as cholesterol, fasting glucose, and arrhythmias. Final eligibility and randomization occur after the run-in and baseline medical assessments because the baseline examinations may reveal participants who meet exclusion criteria not discovered at the preliminary screening. Participants who do not attend the required run-in sessions and baseline examinations within a 5-wk period are excluded for poor adherence. The randomization sequence is computer generated by the study statistician. The sequence is determined from randomly permuted blocks of equal length with fixed numbers of treatment allotments each, to balance treatment enrollments over time. The entire sequence is archived to provide an audit trail when combined with the participant tracking system. Randomization is implemented with treatment assignment letters placed into sequentially numbered, opaque envelopes sealed by the statistician. At randomization, the envelopes are opened in order by a health educator independent of the statistical staff.

Outcome measures and methods. Primary outcome variables are $\dot{V}O_{2max}$ and resting SBP. Secondary outcome variables include fasting blood lipids and lipoproteins (total, HDL- and LDL-cholesterol, and triglycerides), high sensitive C-reactive protein, fasting glucose, anthropometry (body composition and regional fat distribution), BP response to exercise, HR variability, health-related quality of life and other psychosocial variables, and CVD risk determined by a multiple logistic risk function. Other variables of interest include dietary habits, physical activity history, smoking, alcohol intake, sleep habits, medication use (including HRT), demographic characteristics, unstructured physical activity, menstrual history, and personal and family medical history.

The baseline assessments are divided into three sessions. During the first session, each participant rests for 30 min during which time R-R interval variability is recorded. Immediately after this, a minimum of four BP measurements, taken 2 min apart, are obtained using a Colin STBP-780 automated BP unit. Upon completion of the BP measurements, blood is drawn through an antecubital vein. Body composition measurements obtained at the first baseline examination include circumferences of the waist, hip, and upper arm. Skinfold measurements include biceps, triceps, mid-axillary, subscapular, abdominal, suprailiac, thigh, and calf. Lastly, participants undergo a brief screening examination performed either by a physician or nurse. During the second and third assessments, participants complete maximal cycle ergometer tests.

All exercise testing is conducted using a Lode Excalibur Sport cycle ergometer (Groningen, The Netherlands), an electronic, rate-independent ergometer. Subjects are required to practice on the cycle ergometer during the initial baseline medical assessment, providing they meet eligibility requirements. Before the exercise test, a resting electrocardiogram (ECG) is taken and resting HR, SBP, and DBP are measured. Subjects exercise at 30 W for 2 min, 50 W for 4 min, followed by increases of 20 W every 2 min until exhaustion. BP and HR from the ECG are measured at the end of each 2-min stage. During the entire protocol, gas exchange variables ($\dot{V}O_2$, CO_2 production, ventilation, and respiratory exchange ratio [RER]) are recorded every 15 s. During each exercise test, a maximal exercise effort to elicit either $\dot{V}O_{2max}$ or $\dot{V}O_{2peak}$ is used to screen for abnormal BP and ECG responses, as well as determining subsequent exercise prescriptions. The criteria for achieving $\dot{V}O_{2max}$ are RER $>$1.1, a plateau in $\dot{V}O_2$ (change of $<$100 mL·min^{-1} in the last three consecutive 15-s stages), and a HR within 10 beats·min^{-1} of the maximal level predicted by age. Those participants who do not meet these criteria will be classified as having reached their $\dot{V}O_{2peak}$, and subsequent data analysis will account for this factor. After reviewing the results of the first test, participants without medical problems that exclude them from further participation are tested once more to maximum on a separate day.

As before, subjects will exercise at 30 W for 2 min. They will then exercise at 50 W for 4 min to allow collection of steady-state data, followed by increases of 20 W every 2 min until volitional fatigue or exhaustion. Although it may be argued that higher "maximal" values would be obtained on a treadmill, the cycle ergometer gives highly reproducible results ($r = 0.96–0.98$ in the HERITAGE Family Study (27)). In addition to the fact that cycle ergometry is one of the forms of training, it is more tolerable in this population, BP is easier to measure, it requires fewer personnel, and it may have lower risk of injury.

During both exercise tests, respiratory gases are measured using a Parvomedics True Max 2400 Metabolic Measurement Cart. Volume and gas calibrations are conducted before each test. SBP and DBP are measured at rest and during the last 30 s at each power output using a Colin STBP-780 automated BP unit, which is manually calibrated each day. HR is measured directly from the ECG monitoring system. Ratings of perceived exertion (RPE) are obtained using the Borg scale.

Testing at baseline (preintervention) and at 6 months (postintervention) use the same protocols; therefore, HR variability, BP, blood draw, body composition, and two cycle ergometer tests are measured at baseline and 6 months. At baseline and 6 months, the $\dot{V}O_{2max}$ data are averaged for statistical analyses. Variables measured by self-report at baseline, such as dietary habits, physical activity history, smoking, alcohol intake, sleep habits, medication use (including HRT), demographic characteristics, menstrual history, and personal and family medical history, are also measured at 6 months. In addition, unstructured physical activity is monitored on a monthly basis. All randomized participants wear a step counter (Accusplit Eagle, Japan) to record their daily steps and will write comments

Copyright © American College of Sports Medicine. Unauthorized reproduction of this article is prohibited.

on an activity log. Each month, the activity log is returned to the exercise staff. Exercisers will remove the step counter provided at randomization during supervised exercise sessions and will put on a step counter set at zero so that exercise steps and daily steps are recorded separately.

Exercise program rationale and selection of doses. Several different methods have been used to specify and quantify exercise guidelines and physical activity recommendations. Most approaches use the components of exercise intensity, duration of exercise sessions, and frequency of exercise sessions per week to describe and define the overall exercise dose. Typically, ranges for each of these components are given to allow flexibility in combining the components to produce a weekly dose of exercise. For example, the most recent guidelines from the American College of Sports Medicine specify a frequency of 3–5 $d \cdot wk^{-1}$, an intensity of 40–85% $\dot{V}O_{2max}$, and a duration of 20–60 min per session (2). Recent consensus recommendations for physical activity from the U.S. Public Health Service consistently recommend 30 min of moderate-intensity physical activity on most, preferably all, days of the week (23,24,30). Recommendations in these reports for exercise intensity sometimes are presented relative to the person's capacity (e.g., 40–60% $\dot{V}O_{2max}$) as absolute intensity (3 to 6 times resting metabolic rate (RMR) (24)), or in more qualitative terms (brisk walking equivalent to a 15 to 20 $min \cdot mile^{-1}$ pace (23)).

The recommendation that sedentary adults obtain 30 min of moderate-intensity physical activity on most, preferably all, days of the week is the basis for the specific exercise doses to be tested in DREW. Healthy women with similar age, activity pattern, and health status as those in DREW have a $\dot{V}O_{2max}$ of about 6.3 METs (1 MET = RMR or a $\dot{V}O_2$ of 3.5 $mL \cdot kg^{-1} \cdot min^{-1}$) (16,17). This level of cardiorespiratory fitness also is comparable to the cut point defining low fitness of similar aged women of the more than 7000 women participating in the Aerobics Center Longitudinal Study who completed a maximal exercise test. In that study, low-fit women had a substantially higher risk of all-cause and CVD mortality during an 8-yr follow-up than women who were at least moderately fit (7). Adjusted relative risks for low cardiorespiratory fitness were 2.8 for CVD mortality and 2.2 for all-cause mortality.

Women with a $\dot{V}O_{2max}$ of about 6.3 METs who follow the recommendation of 30 min of moderate-intensity physical activity (defined as 50% of 6.3 METs, or 3.2 METs) per day will expend about 1.6 $kcal \cdot kg^{-1}$ for each exercise session (3.2 METs 0.5 h = 1.6) (1,23,24,30). The public health recommendation is that this amount of exercise be obtained on most days of the week (23,24). This realistically refers to 5 $d \cdot wk^{-1}$, which gives an exercise dose of approximately 8 KKW. Therefore, 8 KKW was selected as a dose of energy expenditure that is a reasonable quantitative representation of the recent public health recommendations for sedentary adults. This dose of 8 KKW has produced changes in $\dot{V}O_{2max}$ that placed about 70% of the initially sedentary women above the cut point for low fitness, as defined in the Aerobics Center Longitudinal Study (7).

A major objective of DREW is to evaluate exercise levels 50% above and 50% below the current public health recommendations to test whether the lower dose provides any benefit and whether the higher dose provides more benefit than the standard 8-KKW exercise level. Thus, women will be assigned to either a usual-care control group or to groups that expend 4, 8, or 12 KKW during DREW exercise sessions.

Although the recommendation of 8 KKW is based on accumulating calories over 5 d each week, there was concern that 5 $d \cdot wk^{-1}$ would be an excessive burden and might have an adverse effect on adherence to the exercise intervention program. Studies on exercise frequency show little difference in physiological changes for 3 or more days per week provided the total weekly exercise dose is held constant (2); therefore, women in DREW are asked to obtain their weekly exercise dose in three or four sessions.

Because the various public health pronouncements recommend moderate-intensity exercise for sedentary individuals, the intensity selected for DREW was 50% of $\dot{V}O_{2max}$ across all exercise groups. Moderate-intensity exercise may also be associated with better adherence in initially unfit women, while minimizing dropouts and adverse events. Many studies show that an intensity of 50% $\dot{V}O_{2max}$ is sufficient to produce clinically significant physiological adaptations in sedentary individuals (7,23,24,30). Although it would have been useful to compare various exercise intensities, sample size calculations revealed that the number of subjects needed to test even two exercise intensities was beyond the scope of what could be accomplished within DREW.

There were also discussions on the length of the trial. It has been shown that substantial physiological adaptations occur within the first 3 to 4 months of exercise. Available data suggest that major changes are unlikely beyond 6 months of training (4,26). In view of the expectation of little further adaptation after 6 months and considering the increased logistical and participant burdens (perhaps leading to poorer adherence) and costs of running a highly controlled, laboratory-based study for a longer period, the DREW exercise intervention was limited to 6 months.

Exercise training program. After the average $\dot{V}O_{2max}$ from two cycle ergometer tests on separate days has been calculated, participants are randomly assigned into one of three exercise groups or a nonexercise control group. Each woman in an exercise group alternates training sessions on a recumbent cycle ergometer or treadmill using a standard ramping exercise protocol. The target training intensity is the HR that is associated with 50% of each woman's $\dot{V}O_{2max}$ and the frequency of exercise is 3 or 4× wk^{-1}. The exercise laboratory is open 12.5 $h \cdot d^{-1}$, 5 $d \cdot wk^{-1}$ to allow maximal scheduling convenience.

A computer-controlled exercise training management system allows for input of relevant data points on each woman (week of exercise, KKW dose according to group assignment, HR associated with 50% $\dot{V}O_{2max}$, training HR zone, body weight, and number of visits per week). The computer then provides the appropriate power output (PO)

Copyright © American College of Sports Medicine. Unauthorized reproduction of this article is prohibited.

Participant								
4kcal	Rx	Actual	*8kcal*	Rx	Actual	*12kcal*	Rx	Actual
Week 1	309.6	309.6		406.4	414.7		383.2	384.4
2	308	423.5		515.4	455.1		482.4	482.4
3	312.8	430.1		613	667.5		582.3	585.2
4	315.2	433.4		709.5	710.2		675.4	677.8
5	312.8	312.9		803.6	803.8		761.8	515.9
6	313.6	313.7		809.1	654.3		857.1	928.5
7	313.6	313.6		808.2	958.2		956.8	1028.5
8	313.6	313.6		813.6	816.2		1047.5	1047.6
9	313.6	0		803.6	723.9		1133.2	1061.4
10	317.6	317.6		808.2	909.3		1131.8	1161.8
11	320.8	401		803.6	804.6		1153.7	1253.3
12	320.8	0		809.1	810.8		1146.8	1146.8
13	319.2	399		803.6	803.2		1134.6	1134.6
14	320	400		800	800		1153.2	814.7
15	316.8	401.8		807.3	807.4		1129.1	1217.7
16	311.2	307.4		800	800		1144.1	1196.1
17	309.6	313.4		800	800		1141.3	1284
18	310.4	309.2		796.4	796.4		1145.4	1241
19	308	314.2		800	800		1140	895.3
20	311.2	308.7		798.4	798.4		1144.8	1287.8
21	308.8	304.1		798.4	798.4		1152	1248
22	307.2	272.3		798.4	801.4		1154.4	1154.4
23	304.8	301.4		792	764		1156.8	1160.5
24	300.8	302.5		795.2	795.2		1159.2	1159.2
Total	**7500.0**	**7503.0**		**18293.0**	**18293.0**		**24066.9**	**24066.9**
Diff	3.0			0			0	
Wks left	0			0			0	
Cal/wk	-------			-------			-------	
Cal/sess	-------			-------			-------	
Sess/wk	66	24		87	24		91	24
Avg visits	2.8			3.6			3.8	

FIGURE 2—Weekly tracking report illustrating prescribed weekly caloric expenditure, actual caloric expenditure, and the average number of exercise sessions.

for the cycle ergometer, and the correct speed and grade for the treadmill that will elicit the programmed HR. Knowing the exact PO for the cycle ergometer and the treadmill, the total kilocalories expended each minute and the time needed to reach the target energy expenditure for the exercise session or for the week can be calculated. The duration of each individual session depends on the number of visits required to reach the target KKW.

To monitor the participant's caloric expenditure over the course of 6 months, a weekly tracking report is created (Fig. 2). The weekly report is used to track and calculate caloric adjustments for each of the 24 wk. This report summarizes the prescribed dose of caloric expenditure (group KKW dose multiplied by weight in kilograms) and the actual calories expended per week. The number of calories expended per session is adjusted each week, within the limits of the study design so that the total number of calories expended is equal to the total number prescribed per week of the 24-wk program. This report also averages the number of visits per week so that we can determine whether the participants are exercising at least two, but no more than four, sessions each week.

The training program consists of a 2-min warm-up at a progressively increasing PO until the training HR is reached, after which participants exercise at the appropriate PO and for the appropriate duration. If participants require a recovery or cool-down period, PO is gradually reduced. During exercise, HR is monitored every 3–6 min using a Polar HR monitor (Polar Vantage XL and Polar Vantage NV). The target HR is used to monitor each woman's performance during each session to ensure that she is exercising at the proper intensity. Monitoring HR allows us to control exercise intensity and document the specific amount of exercise done during each session. If the average training HR rises or drops 5 bpm or more during two consecutive weeks, training supervisors will decide to increase or decrease PO during the next training session until the HR associated with the desired percent $\dot{V}O_{2max}$ is attained. This then becomes the new training PO until another HR response is achieved. As women improve their fitness, they will work at a higher PO to reach the required HR and will take less time to expend the required KKW; this could be an incentive to adhere, as less time is needed to reach the weekly goal of 4, 8, or 12 KKW.

Although each participant should expend the KKW required by her group assignment, participants may not be immediately capable of exercising at their required dose. For this reason, there is a gradual progression to the assigned exercise dose (Fig. 3). All exercisers will expend 4 KKW during the first week. Those assigned to the 4-KKW group will remain at that level for 6 months. All other exercisers will increase energy expenditure by 1 KKW each week until they reach the level required for their group. Thus, participants in the 8-KKW group will reach and maintain their dose at week 5 and participants in the 12-KKW group will do the same at week 9. This gradual increase in total energy expenditure should make it easier for women to reach their goal and is expected to minimize fatigue, soreness, injuries, and dropout.

Some flexibility has been built into the program to enhance adherence. Although subjects are expected to train 3 or $4\times$ wk^{-1}, there may be weeks when they can train only two times and would like to exercise longer each session to maintain the same total energy expenditure for the week. This option is limited to participants in the 4-KKW group. In other situations, women may prefer to exercise shorter periods each day over 5 d. This option is limited to participants in the 8- and 12-KKW groups. Participants are not allowed to exercise $<2\times$ wk^{-1} or $>5\times$ wk^{-1}. Women are contacted when they miss a scheduled session so that arrangements can be made to bring them back on schedule as soon as possible. In anticipation of business or family commitments, participants will be allowed to add 1 to 1.5 KKW to their normal dose of exercise for as many weeks as it

Copyright © American College of Sports Medicine. Unauthorized reproduction of this article is prohibited.

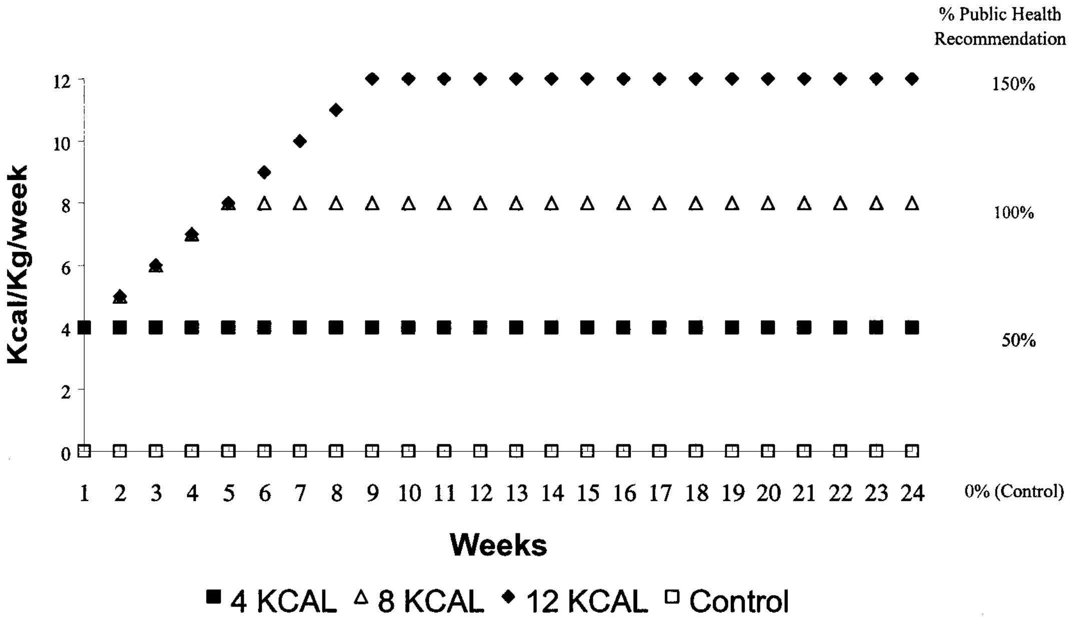

FIGURE 3—Progression of caloric expenditure for the study intervention.

might take to make up or get ahead by 1 wk. These procedures allow for some flexibility but still focus on attaining the target total energy expenditure over the entire 6-month training period.

Adherence. Poor adherence could jeopardize the internal validity (e.g., differential attrition across treatment groups), the generalizability of the findings, or the ability to determine treatment effects. Although ethical considerations require that participants be allowed to withdraw at any time, every possible research-based strategy is being used to minimize dropouts and to maximize the number of women completing the recruitment, run-in, testing, and training phases (10,19). Given that it is unrealistic to expect 100% adherence to the exercise protocol, acceptable adherence is defined as completing 90% or more of the total amount of exercise prescribed. This 90% adherence rate averages out to missing approximately 2.6 wk or 10 sessions.

Unforeseen circumstances might prevent an individual from coming to the center but might still allow her to exercise. In these situations, subjects are given a Polar HR monitor, watch, and instructions on how to operate the watch. They are also asked to complete a log sheet to record the type of aerobic activity completed, duration of exercise, pedometer count, and distance traveled during exercise, if applicable. Data from the HR monitor are then downloaded to confirm the diary information. A total of 16 unsupervised exercise sessions are allowed throughout the study. This provides time for vacations, illnesses, and other commitments that might interfere with a participant's availability to travel to The Cooper Institute for an exercise session. If a participant has not completed 90% of the training by the end of 24 wk but can reach 90% within five training sessions, she will be asked to continue training for no more than two additional weeks.

Each woman will be given up to $500 as an incentive to complete the study. If participants in the control group complete baseline and 6-month measurements, they will receive the entire $500. Exercise group members also have to meet the 90% target adherence rate described earlier, in addition to completing the baseline and 6-month measurements, to receive the entire $500. Payment will be reduced by $50 for each week of missed sessions beyond the 90% adherence target. The maximum deduction for failing to exercise will be $350. Thus, a woman in an exercise group who does not come in to exercise at all will still receive $150 for completing the baseline and 6-month examination. Although this incentive is a substantial amount, it was considered appropriate because the study objective is to evaluate the dose-response effects of exercise. For this reason, excellent adherence to both intervention and measurement is necessary. If DREW were testing the effectiveness of an exercise intervention as a public health strategy, such a payment would not be appropriate. However, DREW is not testing whether financial incentives encourage individuals to exercise; rather, it is evaluating specific responses to various doses of exercise.

Statistical analysis and power. A conservative approach to sample size estimation was employed, and somewhat larger standard deviations than those observed in similar studies were assumed. In addition, there were conservative estimates of effect sizes, allowance for dropouts and partial adherence at higher rates than that achieved in prior studies, and a placebo effect allowed for possible drift between groups. Power calculations were made for the primary outcome measures of $\dot{V}O_{2max}$ and SBP. It was determined that adequate power would be achieved with 150, 100, and 100 women exercising at 4, 8, and 12 KKW, respectively, and with 100 women in the nonexercising control group.

All power computations assumed that intention-to-treat comparisons of baseline to 6-month change scores will be tested at the 5% significance level. Comparison of each

Copyright © American College of Sports Medicine. Unauthorized reproduction of this article is prohibited.

exercise group with the control group was based on the two-sample *t*-test. Tests for a dose-response trend were based on linear regression of change scores across the three exercise doses (controls excluded). Power for the dose-response trend analysis was simulated with 1600 Monte-Carlo replicates. All power computations allowed for 10% dropouts over 6 months, and for 15% partial adherers who would gain only half the exercise benefits of full adherers. Primary outcomes among controls were assumed to change by 1% compared with baseline. We anticipated increases of 7%, 12%, and 15% in $\dot{V}O_{2max}$ at 6 months across the three increasing dose levels, from a baseline level of 1500 $mL \cdot min^{-1}$, and a change-score SD of 200 $mL \cdot min^{-1}$. We also anticipated reductions of 5, 7, and 9 mm Hg in systolic BP at 6 months across the three increasing dose levels and a change score SD of 9 mm Hg. With these parameters, we computed the statistical power to be 0.85, 0.99, and 0.99, respectively, for significant increases in $\dot{V}O_{2max}$ in the groups exercising at 4, 8, and 12 $kcal \cdot kg^{-1} \cdot wk^{-1}$, respectively, compared with the control group. The test for a significant dose-response trend across the three exercise groups has power 0.97. We also computed the statistical power to be 0.84, 0.98, and 0.99, respectively, for significant reductions in systolic BP in those exercising at 4, 8, and 12 $kcal \cdot kg^{-1} \cdot wk^{-1}$, respectively, compared with controls. The test for a significant dose response trend across the three exercise groups has power 0.85.

Out of concern for Type I error rates in performing repeated statistical tests, only two primary outcome measures are prespecified. Other outcomes are categorized as "secondary" or "exploratory," and findings will be interpreted cautiously. Analyses of primary endpoints will follow the intention-to-treat principle. Secondary and exploratory analyses will focus on the secondary endpoints and subgroups. All analyses will take into account prespecified covariates, including age, BMI, HRT status, and baseline values of outcome measures. Analyses of continuous outcome measures will be based on ANCOVA models of 6-month change scores since baseline, and treatment effects will be summarized as least-squares adjusted means (13). Analyses of dose-response trends will be based on ordinary least-squares regression of endpoints across the three exercise groups. Model diagnostics such as quantile plots of Studentized residuals, component-plus-residual plots, and examination of leverage points and outliers will be performed (3). Data transformations of skewed variables may be explored and some analyses may be performed nonparametrically. Analyses of binary outcomes will be based on logistic regression, and analyses of ordered polytomous outcomes will be based on ordered logit proportional-odds models (9). The potential effects of missing data will be explored under various models for nonignorable missing data mechanisms, and through multiple imputation models under ignorable missing data assumptions (18).

SUMMARY

DREW will evaluate the effects of the consensus dose of weekly exercise specified in recent public health recommendations for physical activity on outcome measures related to CVD (14,23,24,30). This study also includes doses of energy expenditure 50% lower and 50% higher than the consensus dose, and will allow the quantification of possible dose-response relations between exercise training and study outcomes.

DREW is an adequately powered, randomized, and controlled trial to test various exercise doses in postmenopausal women who are at moderately elevated risk for CVD and other chronic diseases because of inactivity, overweight, or obesity, and normal or mildly elevated BP. The focus is on evaluating the effect of three exercise doses on cardiorespiratory fitness and SBP (well-established risk predictors for CVD), as well as on several important secondary outcomes. An important strength of the study is the strict control and quantification of the supervised exercise training program. DREW can make important contributions to our understanding of the effects of physical activity in postmenopausal women and help refine public health and clinical recommendations for this group. Postmenopausal women are the most sedentary demographic group in the United States and developing evidence-based physical activity recommendations for them should be given a high priority on the national public health agenda.

This research was supported by the National Heart, Lung, and Blood Institute grant HL66262, a grant from the American Heart Association, and an equipment grant from LifeFitness.

REFERENCES

1. American College of Sports Medicine. *ACSM's Guidelines for Exercise Testing and Prescription*. Media, PA: Williams & Wilkins, 1995, pp. 153–170.
2. American College of Sports Medicine. Position stand: the recommended quantity and quality of exercise for developing and maintaining cardiorespiratory and muscular fitness, and flexibility in healthy adults. *Med. Sci. Sports Exerc.* 30:975–991, 1998.
3. Atkinson, A. C. *Plots, Transformations and Regression: An Introduction to Graphical Methods of Diagnostic Regression Analysis.* New York: Oxford University Press, 1985, pp. 62–79.
4. Badenhop, D. T., P. A. Cleary, S. F. Schaal, E. L. Fox, and R. L. Bartels. Physiological adjustments to higher- or lower-intensity exercise in elders. *Med. Sci. Sports Exerc.* 15:496–502, 1983.
5. Blair, S. N. Exercise prescription for health. *Quest* 47:338–353, 1995.
6. Blair, S. N., W. L. Haskell, P. Ho, et al. Assessment of habitual physical activity by seven-day recall in a community survey and controlled experiments. *Am. J. Epidemiol.* 122:794–804, 1985.
7. Blair, S. N., J. B. Kampert, H. W. Kohl, et al. Influences of cardiorespiratory fitness and other precursors on cardiovascular disease and all-cause mortality in men and women. *JAMA* 276: 205–210, 1996.
8. Clark, L. T., M. M. Karve, K. T. Rones, B. Chang-Demoranville, S. Atluri, and J. G. Feldman. Obesity, distribution of body fat and coronary artery disease in black women. *Am. J. Cardiol.* 73:895–896, 1994.
9. Collet, D. *Modelling Binary Data.* New York: Chapman & Hall, 1991, pp. 43–74.

Copyright © American College of Sports Medicine. Unauthorized reproduction of this article is prohibited.

10. DISHMAN, R. K. *Exercise Adherence: Its Impact on Public Health.* Champaign, IL: Human Kinetics, 1988, pp. 279–396.
11. EXECUTIVE SUMMARY. The Third Report of the National Cholesterol Education Program (NCEP) Expert Panel on Detection, Evaluation, and Treatment of High Blood Cholesterol In Adults (Adult Treatment Panel III). *JAMA* 285:2486–2497, 2001.
12. FLEGAL, K. M., M. D. CARROLL, C. L. OGDEN, and C. L. JOHNSON. Prevalence, and trends in obesity among US adults: 2000. *JAMA* 288:1723–1727, 2002, 1999.
13. FLEISS, J. L. *The Design and Analysis of Clinical Experiments.* New York: John Wiley & Sons, 1986, pp. 186–200.
14. FLETCHER, G. F., G. BALADY, S. N. BLAIR, et al. Statement on exercise: benefits and recommendations for physical activity programs for all Americans: a statement for health professionals by the Committee on Exercise and Cardiac Rehabilitation of the Council on Clinical Cardiology, American Heart Association. *Circulation* 94:857–862, 1996.
15. HADDOCK, B. L., H. P. HOPP, J. J. MASON, G. BLIX, and S. N. BLAIR. Cardiorespiratory fitness and cardiovascular disease risk factors in postmenopausal women. *Med. Sci. Sports Exerc.* 30:893–898, 1998.
16. KING, A. C., W. L. HASKELL, C. B. TAYLOR, H. C. KRAEMER, and R. F. DEBUSK. Group vs. home-based exercise training in healthy older men and women: A community-based clinical trial. *JAMA* 266:1535–1542, 1991.
17. KOHL, H. W., III, A. L. DUNN, B. H. MARCUS, and S. N. BLAIR. A randomized trial of physical activity interventions: Design and baseline data from Project Active. *Med. Sci. Sports Exerc.* 30: 275–283, 1998.
18. LITTLE, R. J. A., and D. B. RUBIN. *Statistical Analysis with Missing Data.* New York: John Wiley & Sons, 1987, pp. 244–265.
19. MARTIN, J. E., and P. M. DUBBERT. Exercise applications and promotion in behavioral medicine: Current status and future directions. *J. Consult. Clin. Psych* 50:1004–1017, 1982.
20. MULLEN, P., and G. R. TABAK. Patterns of counseling techniques used by family practice physicians for smoking, weight, exercise, and stress. *Med. Care* 27:694–704, 1989.
21. NATIONAL INSTITUTES OF HEALTH. *The Sixth Report of the Joint National Committee on Prevention, Detection, Evaluation, and Treatment of High Blood Pressure*, National Institutes of Health. (Eds.). Bethesda, MD: NIH Publication, 1997, pp. 1–70.
22. NATIONAL INSTITUTES OF HEALTH AND NATIONAL HEART LUNG AND BLOOD INSTITUTE. *Clinical Guidelines on the Identification, Evaluation, and Treatment of Overweight and Obesity in Adults: The Evidence Report*, National Institutes of Health and National Heart Lung and Blood Institute. (Eds.). Rockville, MD, 1998, pp. 1–228.
23. NIH CONSENSUS DEVELOPMENT PANEL ON PHYSICAL ACTIVITY AND CARDIOVASCULAR HEALTH. Physical activity and cardiovascular health. *JAMA* 276:241–246, 1996.
24. PATE, R. R., M. PRATT, S. N. BLAIR, et al. Physical activity and public health: a recommendation from the Centers for Disease Control and Prevention and the American College of Sports Medicine. *JAMA* 273:402–407, 1995.
25. PERLOFF, D., C. GRIM, J. FLACK, et al. *Human Blood Pressure Determination by Sphygmomanometry*. Dallas: American Heart Association, 2001, pp. 8–17.
26. SIDNEY, K. H., and R. J. SHEPHARD. Frequency and intensity of exercise training for elderly subjects. *Med. Sci. Sports* 10:125–131, 1978.
27. SKINNER, J. S., K. M. WILMORE, A. JASKOLSKA, et al. Reproducibility of maximal exercise test data in the HERITAGE Family Study. *Med. Sci. Sports Exerc.* 31:1623–1628, 1999.
28. SPEROFF, L. A signal for the future. In: *Treatment of the Postmenopausal Woman: Basic and Clinical Aspects*, R. A. Lobo (Ed.). New York: Raven Press, 1994, pp. 1–8.
29. THE EXPERT COMMITTEE ON THE DIAGNOSIS AND CLASSIFICATION OF DIABETES MELLITUS. Report of the Expert Committee on the diagnosis and classification of diabetes mellitus. *Diabetes Care* 20: 1183–1197, 1997.
30. U.S. DEPARTMENT OF HEALTH AND HUMAN SERVICES. Physical activity and health: a report of the Surgeon General. Atlanta, GA: U.S. Department of Health and Human Services, Centers for Disease Control and Prevention, National Center for Chronic Disease Prevention and Health Promotion, 1996, pp. 9–208.

Copyright © American College of Sports Medicine. Unauthorized reproduction of this article is prohibited.
